# The mutation T477A in HIV-1 reverse transcriptase (RT) restores normal proteolytic processing of RT in virus with Gag-Pol mutated in the p51-RNH cleavage site

**DOI:** 10.1186/1742-4690-7-6

**Published:** 2010-02-01

**Authors:** Michael E Abram, Stefan G Sarafianos, Michael A Parniak

**Affiliations:** 1 HIV Drug Resistance Program, National Cancer Institute, Frederick, MD, 21702, USA; 2 University of Missouri-Columbia, Department of Molecular Microbiology and Immunology, Columbia, MO, 65211, USA; 3 University of Pittsburgh School of Medicine, Department of Microbiology and Molecular Genetics, Pittsburgh, PA, 15219, USA

## Abstract

**Background:**

The p51 subunit of the HIV-1 reverse transcriptase (RT) p66/p51 heterodimer arises from proteolytic cleavage of the RT p66 subunit C-terminal ribonuclease H (RNH) domain during virus maturation. Our previous work showed that mutations in the RT p51↓RNH cleavage site resulted in virus with defects in proteolytic processing of RT and significantly attenuated infectivity. In some cases, virus fitness was restored after repeated passage of mutant viruses, due to reversion of the mutated sequences to wild-type. However, in one case, the recovered virus retained the mutated p51↓RNH cleavage site but also developed an additional mutation, T477A, distal to the cleavage site. In this study we have characterized in detail the impact of the T477A mutation on intravirion processing of RT.

**Results:**

While the T477A mutation arose during serial passage only with the F440V mutant background, introduction of this substitution into a variety of RT p51↓RNH cleavage site lethal mutant backgrounds was able to restore substantial infectivity and normal RT processing to these mutants. T477A had no phenotypic effect on wild-type HIV-1. We also evaluated the impact of T477A on the kinetics of intravirion Gag-Pol polyprotein processing of p51↓RNH cleavage site mutants using the protease inhibitor ritonavir. Early processing intermediates accumulated in p51↓RNH cleavage site mutant viruses, whereas introduction of T477A promoted the completion of processing and formation of the fully processed RT p66/p51 heterodimer.

**Conclusions:**

This work highlights the extraordinary plasticity of HIV-1 in adapting to seemingly lethal mutations that prevent RT heterodimer formation during virion polyprotein maturation. The ability of T477A to restore RT heterodimer formation and thus intravirion stability of the enzyme may arise from increased conformation flexibility in the RT p51↓RNH cleavage site region, due to loss of a hydrogen bond associated with the normal threonine residue, thereby enabling proteolytic cleavage near the normal RT p51↓RNH cleavage site.

## Background

Human immunodeficiency virus type 1 (HIV-1) reverse transcriptase (RT) is a multifunctional viral enzyme that catalyzes all chemical steps in the conversion of HIV-1 genomic RNA into double stranded viral DNA. While the RT gene encodes a polypeptide of 66 kDa (translated as a part of a much larger 160 kDa Gag-Pol polyprotein), RT in infectious virions is a heterodimer of 66 kDa (p66) and 51 kDa (p51) subunits [1]. The latter subunit, p51, is derived from the larger p66 subunit (or a larger RT precursor) by HIV-1 protease (PR)-catalyzed cleavage of the p51↓RNH junction during viral maturation. This event results in the removal of a 15 kDa C-terminal ribonuclease H (RNH) domain [2-5]. The tertiary folding of each subunit in RT differs, resulting in an asymmetric heterodimer [6,7]. RT catalytic activities are located in the p66 subunit, whereas the p51 subunit of the RT heterodimer is believed to play primarily a structural role [8-10]. In addition to its catalytic function, the RNH domain of the p66 subunit has been suggested to play a structural role in the maintenance of RT stability [11-16].

Since HIV-1 virions contain essentially equivalent amounts of p66 and p51 RT subunits [17,18], proteolytic cleavage of the p51↓RNH junction may possibly be an important factor in the production of replication-competent virions. Furthermore, both recombinant RT p66/p66 homodimers and RT p66/p51 heterodimers show similar catalytic properties (DNA polymerase and RNH activities) *in vitro *[19-21], which begs the question, why is additional proteolytic cleavage of the p51↓RNH junction needed *in vivo *during virus maturation? We recently showed that mutagenesis of the RT p51↓RNH protease recognition sequence (AETF^440↓ ^Y^441^VDG) resulted in aberrant proteolytic processing producing HIV-1 virions with greatly decreased levels of RT that in many cases was primarily RT p51, leading to substantially reduced replication capacity [22]. We hypothesized that the p51↓RNH cleavage event was essential to confer proteolytic stability to RT. Repeated passage of some of these p51↓RNH cleavage site mutant viruses eventually led to the appearance of relatively normal replication kinetics. These recovered viruses possessed normally processed heterodimeric p66/p51 RT. In some cases, the recovery was due to reversion of the mutant sequence to the normal wild-type p51↓RNH protease recognition sequence. However, in one case, the recovered virus maintained the mutated protease recognition sequence (F440V), but now possessed a single additional amino acid substitution, T477A, distal to the normal the p51↓RNH cleavage site between F440 and Y441 [22].

In the present work we examined in detail the effect of the conservative T477A substitution on alleviating the detrimental phenotypic effect of the F440V mutation in the p51↓RNH protease recognition sequence. Interestingly, the T477A substitution also alleviated the phenotypic impact of many other mutations in the p51↓RNH cleavage region, despite the fact that this compensatory substitution did not normally arise in revertants of these mutant viruses. Furthermore, the T477A compensatory substitution was also effective at restoring infectivity to some p51↓RNH mutants that never recovered infectivity during repeated passage. In all cases, the addition of the T477A substitution resulted in virions containing seemingly wild-type levels of heterodimeric p66/p51 RT despite the continued presence of mutations in the p51↓RNH protease recognition sequence. We propose that when the p51↓RNH junction is mutated, the T477A compensatory substitution may enable HIV-1 PR-mediated proteolytic processing of RT p66 at another site close to the normal proteolytic cleavage point, thereby enabling formation of an RT heterodimer refractory to additional proteolytic degradation.

## Results

### Effect of the RT T477A substitution on infectivity and virion RT content of p51↓RNH cleavage site mutants

**Figure 1:**
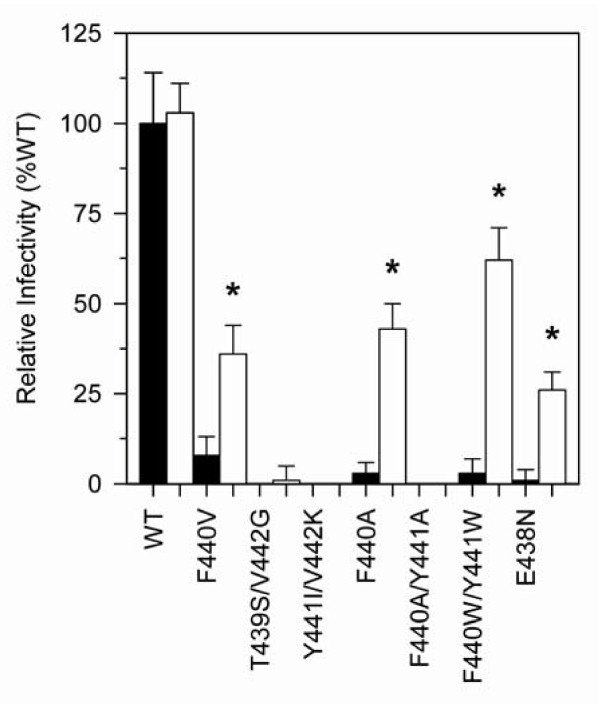
**Infectivity of WT and p51↓RNH ± T477A mutant virus in single cycle replication assays**. HIV-1 derived from transfection of 293T cells was added to P4R5 indicator cells (5 × 10^3^), and infectivity was assessed 48 h post infection as indicated in Materials and Methods. Black and white bars indicate p51↓RNH - T477A or p51↓RNH + T477A mutant viruses respectively, derived from transfected 293T cells, normalized to 25 ng viral p24 at time of infection. Infectivity was determined after 48 h of culture by fluorescent measurement of β-galactosidase gene expression, as described in Materials and Methods. Data are means ± S.D. from 16 independent experiments. Asterisks (*) indicate statistical significance (*p *< 0.001) between -T477A and +T477A mutant viruses, calculated using a one-tailed Student's *t*-test assuming equal variance.

**Figure 2:**
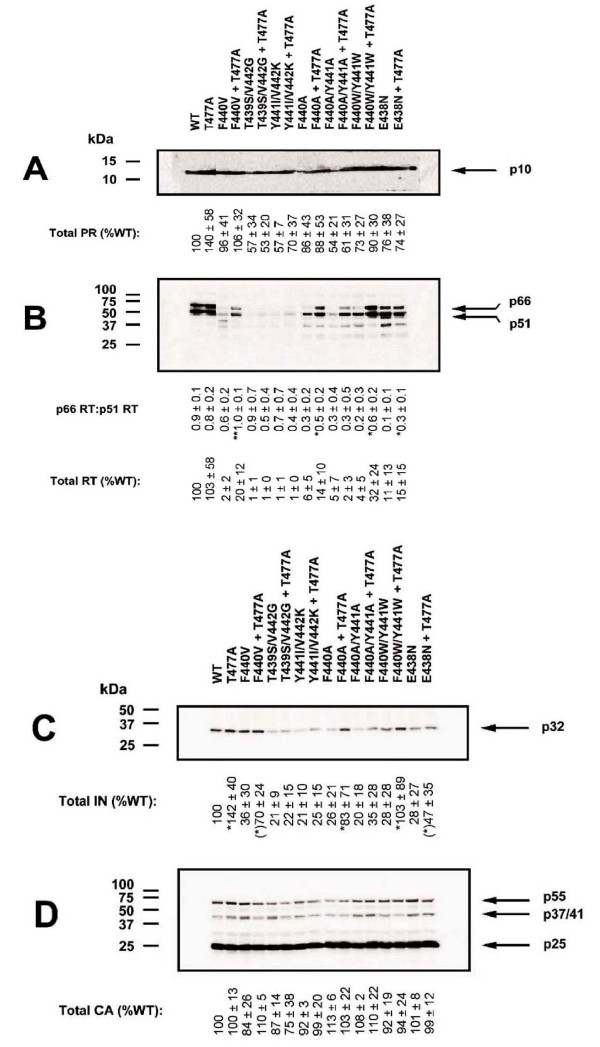
**Effect of p51↓RNH ± T477A mutations on viral particle protein composition**. Western blots of wild-type (WT) and p51↓RNH ± T477A mutant viruses (1 μg viral p24) generated by transfection of 293T cells and probed with (A) anti-PR, (B) anti-RT, (C) anti-IN, and (D) anti-p24 antibodies. The positions of molecular size markers are shown to the left of each panel. Arrows to the right of each panel indicate the positions and molecular masses of immunoreactive viral proteins. The relative mean proportion of p66 RT to p51 RT (p66:p51) and the total viral content of RT, IN and CA were determined from multiple experiments (*n *= 3) by densitometric scanning analysis of ECL-exposed blots under subsaturating conditions. Statistical significance of the T477A compensatory effect was determined for each individual mutant virus relative to its non-substituted counterpart using a one-tailed Student's *t*-test assuming equal variance. Asterisks indicate the degree of statistical significance in relation to the size of the type I error: (*)*p *< 0.10, *p < 0.05, **p < 0.01. In Figure 2A and 2B, WT and WT+T477A samples (two leftmost lanes) are from a different gel than the rest of the samples, as the number of wells in the electrophoresis apparatus was unable to accommodate all samples simultaneously. However, all electrophoresed samples had the same amount of p24 (see Methods) and were processed simultaneously (using two identical electrophoresis apparatus). Both resultant gels were imaged simultaneously by chemiluminescence as described in Materials and Methods.

In order to validate the role of T477A in the reversion phenotype of F440V, we introduced this amino acid substitution into HIV-1 clones containing a variety of other p51↓RNH cleavage site mutations that we previously showed to be detrimental to proper RT processing, as well as into wild-type HIV-1. Introduction of the T477A mutation into a wild-type HIV-1 background had no effect on virus replication (Fig. 1; Table 1), or on virion Pol protein content (Fig. 2). However, introduction of this substitution into a molecular clone of the F440V p51↓RNH cleavage site mutant HIV-1 resulted in a significant increase in infectivity of this mutant virus (Fig. 1, Table 1) as well as a considerable acceleration in viral spread (data not shown), thereby validating the compensatory nature of the T477A substitution in the context of the F440V p51↓RNH cleavage site mutation. Interestingly, the T477A substitution also significantly improved the infectivity (Fig. 1, Table 1) and replication kinetics of 3 out of 6 other p51↓RNH cleavage site mutants (data not shown) that originally showed severe attenuations in infectivity, despite the fact that this compensatory substitution arose only during passage of the F440V mutant.

**Table 1:** Replication capacity of HIV-1 with mutations in the p51↓RNH cleavage site ± T477A

RT p51↓RNH mutation	**Virus titer (% wild-type control)**^a^		
	- T477A	+ T477A	
WT	100	100 ± 30	(N.S.)^b^
F440V	< 1 ± 1	10 ± 3	(p < 0.01)
T439S/V442G	---^c^	---	
Y441I/V442K	---	---	
F440A	<1 ± 1	10 ± 5	(p < 0.01)
F440A/Y441A	<1 ± 1	1 ± 1	(N.S.)
F440W/Y441W	<1 ± 1	32 ± 14	(p < 0.01)
E438N	<1 ± 1	5 ± 4	(p < 0.5)

^*a *^Infectious virus titer (TCID_50_/ml) was determined in MT-2 cells and was normalized by dividing by the amount of viral p24 (ng/ml), as described in Materials and Methods. Data are presented as mean % wild-type virus titer ± standard deviation from four individual experiments.

^*b *^*P *values were calculated using a one-tailed Student's *t*-test assuming equal variance. N.S., not significant.

^c ^No virus titer noted

The p51↓RNH cleavage site mutants that showed improved infectivity due to the presence of the T477A substitution also showed significantly increased virion levels of RT (Fig. 2B) and integrase (IN) (Fig. 2C) and normalized the ratio of the p66 RT and p51 RT subunit content in most mutant viruses, suggesting near normal proteolytic processing and stability of RT p66 in these mutant viruses (Fig. 2B). The impact of the T477A substitution was not due to increased virion incorporation of Pr160^gag-pol^, as normal levels of PR were present in all mutant viruses (Fig. 2A), and the presence of T477A did not alter virion incorporation of Pr160^gag-pol ^possessing inactive PR (data not shown).

### Effect of the RT T477A substitution on intravirion processing of the Pr160^Gag-Pol ^polyprotein

**Figure 3:**
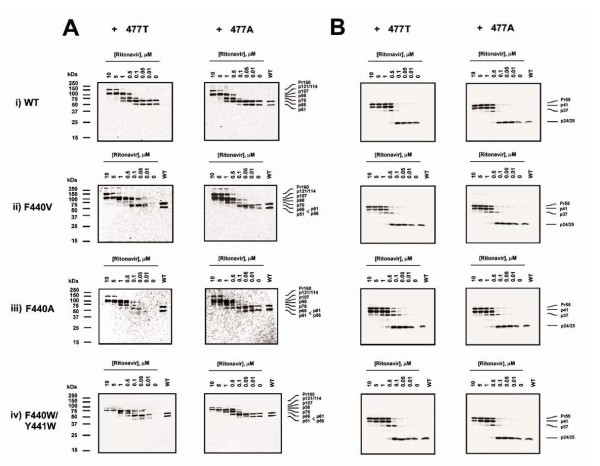
**Effect of p51↓RNH ± T477A mutations on ordered intravirion processing of Gag and Gag-Pol polyproteins**. Virus-containing culture supernatants derived from the transfection of COS-7 cells in the presence of various concentrations of ritonavir were subjected to SDS-10% PAGE resolution and Western blotting analysis. (A) Pr160^Gag-Pol ^and (B) Pr55^gag ^polyprotein processing intermediates were visualized with anti-RT and anti-p24 monoclonal antibodies respectively, followed by ECL exposure. Analyses of p51↓RNH mutant viruses containing the wild-type 477T or the mutant 477A are in the left and right panels, respectively. The positions of molecular size markers are shown to the left of each panel. Lines to the right of each panel indicate the positions and estimated molecular masses of predicted polyprotein processing intermediates [24,27].

To better evaluate the effect of p51↓RNH cleavage site mutations in the absence and in the presence of the T477A substitution on the formation of mature RT p66/p51 heterodimers, we compared the intravirion accumulation of Pr160^Gag-Pol ^polyprotein proteolytic processing intermediates by preparing virions in the presence of increasing concentrations of the HIV-1 PR inhibitor ritonavir (RTV). Wild-type virions with or without the T477A substitution showed similar RTV dose-dependent diminutions in Pr55^Gag ^and Pr160^Gag-Pol ^proteolytic processing as assessed by decreasing levels of RT p66/p51 and Gag p24, and increasing levels of higher molecular weight polyproteins reactive with either RT-specific or Gag-p24-specific antibodies (Fig. 3A and 3B). The apparent masses of these larger processing intermediates are consistent with previous findings and predictions based on protease cleavage sites in the polyproteins [23-26].

Consistent with our previous findings [22], mutations in the p51↓RNH cleavage site resulted in significantly diminished levels of RT p66/p51 in virions produced in the absence of RTV. In fact, with most mutants, p51↓RNH cleavage site mutant virions contained virtually no RT immunoreactive proteins (Fig. 3A, left panel). RT antibody reactive proteins increased substantially in virions produced in the presence of RTV concentrations above 0.1 μM. However, in no case did RTV treatment lead to the appearance of RT p66/p51 in the mutant virions. Instead, the RT antibody immunoreactive proteins corresponded to Gag-Pol polyprotein processing intermediates between 160 and 100 kDa. Addition of the T477A substitution to the p51↓RNH cleavage site mutants resulted in virions with elevated levels of p66 RT at RTV concentrations less than 0.1 μM and p66/p51 RT in the absence of RTV, suggesting relatively normal processing and proteolytic stability of RT (Fig. 3A, right panel). These virions produced in the presence of RTV concentrations above 0.1 μM showed higher molecular weight Gag-Pol polyprotein intermediate profiles similar to those seen in virions lacking the T477A substitution, due to RTV inhibition of normal viral polyprotein processing.

## Discussion

The proteolytic processing of Gag and Gag-Pol polyproteins into their respective structural proteins and functional enzymes is an essential stage in HIV replication. This processing does not occur randomly, but rather appears to comprise some degree of ordered cleavage events to provide functional and infectious virus [23,24,27]. However, the kinetics of and factors defining these cleavage events *in vivo *remain poorly defined. One of the most intriguing polyprotein proteolytic cleavage events is the RT p51↓RNH cleavage needed to form the obligate p66/p51 RT heterodimer. While all three *pol*-derived enzymes (PR, RT, IN) are active only as oligomers, only RT is a heterodimer. We previously showed that mutations introduced into the RT p51↓RNH protease recognition and cleavage site, which we predicted would result in accumulation of unprocessed RT p66, instead resulted in severe attenuations of HIV-1 infectivity due to inappropriate intravirion degradation of RT by the viral protease [22]. Based on these findings, we suggested that the proteolytic cleavage at the RT p51↓RNH junction to form the RT p66/p51 heterodimer was essential to stabilize RT and to prevent extensive intravirion HIV PR-mediated degradation of RT.

HIV has an extraordinary adaptive capacity, and we asked whether virions mutated to prevent RT p51↓RNH cleavage could surmount this barrier to normal phenotypic maturation. We tested this by carrying out long term passage of RT p51↓RNH cleavage site mutants in permissive cells. Three different phenotypes were found, depending on the initial cleave site mutations introduced [22]. Some mutants never recovered replication capacity. Some mutants recovered replication capacity due to reversion of the p51↓RNH cleavage site mutations to wild-type sequences. In some cases however, the p51↓RNH cleavage site mutations remained, but additional amino acid changes in RT were found. The predominant consensus change was the conservative second-site substitution T477A, initially identified in the background of an F440V p51↓RNH cleavage site mutant.

Addition of T477A into the F440V mutant virus resulted in more normal virion RT p66/p51 content and an increase in virion infectivity (Fig. 1). Importantly, addition of this mutation into several other RT p51↓RNH cleavage site mutants also resulted in restoration of virion RT p66/p51 content and infectivity to near wild-type levels. As an example, introduction of T477A in the background of the "lethal" F440W/Y441W mutation resulted in an increase of HIV-1 infectivity from near zero to about 70% of wild-type levels (Fig. 1).

**Figure 4:**
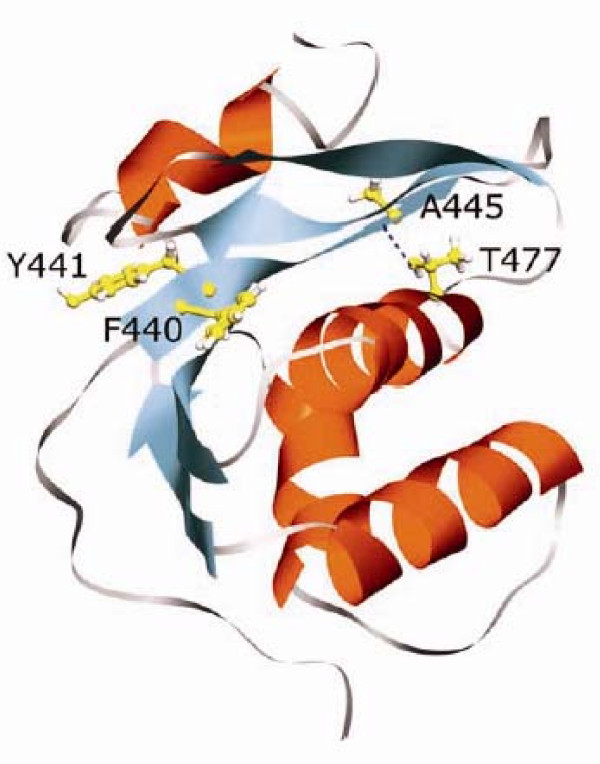
**Positions of the p51↓RNH cleavage site and the residue T477 in HIV-1 RT**. Ribbon diagram of amino acid residues 425-560 depicting the RNH domain of HIV-1 RT, adapted from PDB 1LDO[42]. The H-bond between the hydroxyl of T477 and the main chain of A445 is indicated by a dashed line. Details are provided in the text. The molecular graphics image was produced using the UCSF Chimera package from the resource for Biocomputing, Visualization, and Informatics at the University of California, San Francisco (supported by NIH P41 RR-01081) [43].

The compensatory nature of the T477A second-site mutation highlights the importance of maintaining a proteolytically stable form of RT during virus maturation. The Thr to Ala change at residue 477 is polymorphic, present in about 4% of HIV-1 sequences [28], suggesting that it may confer some advantage or at least is benign under normal replication conditions. This is consistent with the lack of phenotypic effect following introduction of T477A into a wild-type RT background (Fig. 1 and 2). Our studies suggest that virtually any mutation in the PR-recognition sequence defining the p51↓RNH cleavage site (residues 437-444) leads to non-infectious virus, and work by others had indirectly suggested that residue 438 was important for correct RT heterodimer processing [29]. So how can a conservative change such as T477A in the RNH domain of RT possibly alleviate this detrimental phenotype? Proteolytic processing occurs with intermediate forms of RT preceding the final p66/51 heterodimer, and RT structures are available only for the latter. Nonetheless, examination of the RT p66/51 structure enables us to propose the following model. The p51↓RNH cleavage site, defined by residues F440 and Y441, is part of a β 1 sheet that is nicely packed against the α A helix that carries T477. This places the p51↓RNH cleavage site proximal in space to the T477 residue whose side chain OH engages in a hydrogen bond with the main chain of residue A445 (Fig. 4). Despite the extensive hydrophobic interactions between this helix and the β-sheet throughout their length, the hydrogen bond between residues A445 and T477 is the only electrostatic interaction between these secondary structure elements. We surmise that a T477A substitution would eliminate this hydrogen bond, resulting in increased regional flexibility such that proteolytic cleavage occurs at an alternate site despite the continued presence of the p51↓RNH mutations. Such alternate cleavage sites have been previously suggested [5,30], but have yet to be identified directly from virus-derived RT. A comparison of several HIV-1 RT crystal structures provides indirect evidence for the potential flexibility in this region. Specifically, the superposition of the RNH domains of several RT structures shows notable variability in the main chain conformation; and in some cases (e.g., PDB 1FK9[31]), parts of the β-sheet (residues 444 - 454) are missing, possibly due to poor electron density resulting from multiple conformations in this region.

The intravirion RT processing defects imparted by the p51↓RNH cleavage site mutations are similar to those noted with mutations such as W401A [32], a mutation which impacts RT dimerization [33]. It is not inconceivable that the RT p66 monomer would be more proteolytically labile than the dimer, thus mutations that prevent RT dimerization would lead to virions with reduced RT content such as seen with the p51↓RNH cleavage site mutants. Despite the similarity in the phenotype imparted by the W401A and the p51↓RNH cleavage site mutations, we do not think that the latter act by reducing RT dimer formation. The p51↓RNH cleavage site is not involved in significant subunit interface interactions, and as well is quite removed from the Trp motif that plays a major role in RT dimer stabilization.

## Conclusion

In summary, we have demonstrated that both virion infectivity and proteolytic stability of RT with p51↓RNH cleavage site mutations can be restored to various extents by the second-site compensatory mutation T477A. Studies are currently in progress to characterize the C-terminal amino acid sequence of the RT p51 subunit from HIV-1 with p51↓RNH cleavage site mutations and the T477A substitution in RT in order to determine whether this compensatory mutation enables proteolytic cleavage at a position other than the normal F440↓Y441 location.

## Materials and methods

### Reagents

The following reagents were obtained through the AIDS Research and Reference Reagent Program, Division of AIDS, NIAD, NIH: anti-HIV-1_SF2 _p24/25 IgG mAb (76C) from Dr. Kathelyn Steimer, Chiron Corporation, and anti-HIV-1_HXB2 _IN (2C11 and 8G4) IgG mAb from Dr. Dag Helland. Rabbit anti-HIV-1 PR polyclonal serum directed against PR residues 86-108 [34,35] was a generous gift from Dr. Stuart Le Grice, NCI-Frederick (Frederick, MD). Anti-HIV-1_IIIB _RT IgG mAbs specifically reacting with HIV-1 RT were previously generated in our laboratory [36]. Goat anti-mouse-HRP and donkey anti-rabbit secondary mAb were products of GE HealthCare (formerly Amersham Pharmacia Biotech, Piscataway, NJ). The SuperPico ECL Substrate System for detection of peroxidase-labeled antibody was obtained from PIERCE (Rockford, IL). 4-methylumbelliferyl-β-D-galactopyranoside (4-MUG), a fluorescent substrate for β-galactosidase, was obtained from Sigma-Aldrich (St. Louis, MO). HIV-1 p24 antigen ELISA kits were obtained from SAIC-Frederick (Frederick, MD). Sequencing, PCR amplification and mutation-containing oligonucleotide primers were purchased from Invitrogen (Carlsbad, CA).

### Cell lines

The human T-lymphocytoid MT-2 and MT-4 cell lines were maintained in RPMI 1640 supplemented with 10% fetal bovine serum (FBS). Human 293T and monkey COS-7 fibroblast cell lines were maintained in Dulbecco's modified Eagle medium (DMEM) supplemented with 10% FBS. The P4R5 HIV infection indicator cells were obtained from Dr. John Mellors, University of Pittsburgh, and maintained in DMEM/10% FBS supplemented with puromycin (0.5 μg/mL). P4R5 cells express CD4, CXCR4 and CCR5 as well as a β-galactosidase reporter gene under the control of an HIV LTR promoter [37].

### Preparation, cloning and sequencing of p51↓RNH mutant revertants

As described in our previous report [22], MT-2 cells were inoculated with p51↓RNH cleavage site mutant viruses and then maintained in culture for up to 30 d or until cytopathic effects were noted. Virus-containing cell-free culture supernatants were then used to infect fresh MT-2 cells. Cells were isolated 5 d post-infection and then chromosomal DNA was extracted using the QIAamp DNA Mini Kit protocol (Qiagen Inc., Valencia, CA). The HIV-1 RT-encoding region was amplified by PCR and cloned into pCR-T7/CT TOPO (Invitrogen, Carlsbad, CA) for sequencing analysis.

### Mutagenesis of HIV-1 molecular clones and production of recombinant virus

Plasmid pSVC21-BH10 encodes an infectious molecular clone of HIV-1 IIIB (HxB2) and carries an SV40 origin of replication for expression in 293T and COS-7 cells [38]. In our previous study [22], we used pSVC21-BH10 to prepare ten different variants mutated in the RT p51↓RNH cleavage site (amino acid residues 437-443), namely A437I, V442S, F440W, F440V, T439S/V442G, Y441I/V442K, F440A, F440A/Y441A, F440W/Y441W, and E438N. We introduced the mutation T477A into each of these p51↓RNH cleavage site mutants as well as into the wild-type clone using the Quick Change™ Site-Directed Mutagenesis kit protocol (Stratagene, La Jolla, CA). In order to assess the incorporation of the Pr160^Gag-Pol ^polyprotein precursor into recombinant virions, we also prepared a second set of HIV-1 clones containing the D25A inactivating mutation in the PR coding region to prevent proteolytic processing of Pr160^Gag-Pol^. The presence of all mutations were verified by sequencing. Recombinant virus was prepared by transfection of 293T cells using calcium phosphate co-precipitation. Virus-containing culture supernatants were harvested 60 h post-transfection and clarified by centrifugation (3,000×g, 1 h at 4°C). The level of recombinant virus production was quantified by measurement of HIV-1 p24 antigen. Virus preparations were then aliquoted and stored at -80°C until use.

### HIV-1 infectivity assays

Infectivity of virus particles produced by transfection of 293T cells was determined by addition of defined quantities of HIV-1 p24 antigen to target infectable cells. Single-cycle viral infectivity was assessed in 96-well microplate assays using P4R5 cells (5 × 10^3 ^cells/well). Cells were inoculated with 25 ng HIV-1 p24/well and the extent of infection was evaluated 48 h post-infection using a fluorescence-based β-galactosidase detection assay. Briefly, infected cells were washed, then incubated with 100 μL lysis buffer (60 mM Na_2_HPO_4_, 40 mM NaH_2_PO_4 _(pH 7.2), 1 mM MgSO_4_, 100 mM β-mercaptoethanol, 2% [v/v] Triton X-100) for 1 h at 37°C. β-galactosidase activity was assessed by addition of 50 μL 4-MUG to a final concentration of 0.5 mM, incubation for 1 h at 37°C, and then quenched with 150 μL 0.2 M Na_2_CO_3_, pH 11.2. Fluorescence intensity was assessed with a SPECTRAmax GEMINI XS dual-scanning microplate spectrofluorometer (Molecular Devices, Sunnyvale, CA) using an excitation wavelength of 355 nm and an emission wavelength of 480 nm, with cutoff filter set to 475 nm.

Multiple-round viral replication (virus spread) was assessed using MT-2 cells cultured in 96-well microplates (6.5 × 10^4 ^cells/well). Cells were inoculated with 25 ng HIV-1 p24/well. HIV-1 induced cytopathic effects were evaluated daily by microscopic observation of HIV-1 induced syncytium formation (data not shown), as previously described [39,40]. In a separate, but complementing experiment (Table 1), each virus was titered on MT-2 cells to evaluate the median tissue culture infective dose (TCID_50_/ng p24) after seven d post-infection, as described [41].

### Analysis of virion proteins

HIV-1 virions were isolated by centrifugation of aliquots of cell-free culture supernatants (corresponding to 1 μg viral p24) at 175,000×g for 1.5 h at 4°C through a 20% (w/v) sucrose cushion. Pelleted virions were lysed in 16 μL of 20 mM Tris-Cl (pH 8.0) containing 120 mM NaCl, 2 mM EDTA, 0.5% deoxycholate, 0.5% NP-40 (v/v), as well as the protease inhibitors phenylmethyl sulfonyl fluoride (2 μg/mL), aprotinin (10 μg/mL) and pepstatin A (10 μg/mL). Virion protein composition was assessed by Western blotting after resolution of the proteins by 10% SDS-PAGE. Specific viral proteins were detected by incubating the blots with anti-HIV-1 RT mAbs (6 μg/mL), anti-HIV-1 IN (mixed 2C11 and 8G4, 1:40 dilution), anti-HIV-1 PR monospecific antiserum (1:40 dilution) or anti-HIV-1 p24 mAb (3 μg/ml) followed by incubation with the appropriate HRP-conjugated secondary antibody (1:1000 dilution). Non-specific binding was minimized by blocking the blots with 7% (w/v) skim milk/0.05% (v/v) Tween 20 in phosphate buffered saline. Normal goat or donkey serum was added to the blocking solution at a 1:100 (v/v) dilution where appropriate. Immunoreactive protein bands were visualized and quantified by enhanced chemiluminescence (ECL) using a BioRad VersaDoc Imaging System.

### Analysis of intravirion proteolytic processing of Pr55^Gag ^and Pr160^Gag-Pol ^polyproteins

The accumulation of polyprotein intermediates formed during HIV-1 PR-mediated processing of Pr160^Gag-Pol ^in nascent HIV-1 virions was assessed by immunoprobing of Western blots of recombinant virions produced in the presence of varying concentrations of the PR inhibitor ritonavir (RTV). Briefly, COS-7 cells (1.6 × 10^5 ^cells/well) were transfected with 3 μg of proviral plasmid DNA (pSVC21-BH10) using LipofectAMINE Plus (Invitrogen, Carlsbad, CA) for 3 h. The transfection medium was then replaced with cell culture medium containing varying concentrations of RTV. Cell culture supernatants were harvested 48 h post-transfection and HIV-1 virions were isolated by centrifugation at 175,000×g for 1.5 h at 4°C through a 20% (w/v) sucrose cushion. Purified virions were quantified by analysis of HIV-1 p24 antigen, and virion protein composition was assessed after lysis and SDS-PAGE resolution as described above.

## Abbreviations

HIV-1: human immunodeficiency virus type 1; PR: protease; RT: reverse transcriptase; RNH: ribonuclease H; IN: integrase; WT: wild-type; ritonavir: RTV.

## Competing interests

The authors declare that they have no competing interests.

## Authors' contributions

MEA designed the study and carried out most of the experimental procedures and data analysis, and drafted the manuscript. SGS contributed to analysis of the structural basis for the observed phenotype. MAP made substantial contributions to the conception and design of the study, data interpretation, and in preparation of the final manuscript.
